# Early sexual initiation, sexual activity below legal age of consent, and sexual coercion: distinguishing overlapping public health challenges

**DOI:** 10.1016/j.jped.2026.101607

**Published:** 2026-09-17

**Authors:** Venkatraman Chandra-Mouli

**Affiliations:** World Health Organization, Department of Sexual and Reproductive Health and Research, Switzerland

In my editorial commentary I address the Pelotas Birth Cohort Study; the just-published report of a study by da Silveira et al. [1] on sexual initiation, forced sexual initiation and associated factors; the key findings of the study, my interpretation of the findings, and three implications for research and action.

## The Pelotas Birth Cohort Study

Three key strengths of the Pelotas Birth Cohort Study are its longitudinal population-based design, large sample with high retention, and rigorous methods. The 2004 Pelotas Birth Cohort enrolled 4,231 newborns — 99.2% of eligible births in Pelotas — and has followed participants repeatedly from birth through adolescence. Follow-up rates remained remarkably high, declining from 95.7% at three months to 86.6% at 11 years; the 18-year follow-up used in the present study achieved a rate of 85%. The cohort has used standardized data-collection procedures and carefully documented methods throughout its successive follow-ups. These features make the Pelotas cohort an unusually strong platform for examining how experiences and exposures earlier in life relate to health and behavior in adolescence and young adulthood [2,3].

## Key findings from the study

Against this strong methodological backdrop, the study reports on the initiation of sexual activity and the context in which it occurs [1]. At 18 years, 64% of the sample reported having had sexual intercourse, with a slightly higher prevalence among girls. Among those who were sexually active, 15.7% of boys and 11.1% of girls (13.3% overall) reported having had their first sexual intercourse before age 14. Forced first sexual intercourse was reported by 2.6% of girls and 0.5% of boys (1.6% overall). The levels of sexual intercourse before age 14 was higher among boys, those who identified themselves as asexual/other rather than heterosexual, those who were currently using drugs daily or almost daily, those who had a higher number of lifetime sexual partners, and those whose first sexual intercourse was forced. The levels of forced first sexual intercourse was higher among girls, those who described themselves as pansexual, those who had their first sexual intercourse before age 14, and those who reported a higher number of lifetime sexual partners. These associations should not, however, be interpreted as causal.

## Interpreting the study’s findings

In examining first sexual intercourse, and first forced sexual intercourse, it is important to consider whether the context of sexual activity was legal or illegal, and consensual or coerced. Under Brazilian law, persons under 14 years of age cannot legally consent to sexual activity; sexual intercourse or other sexual acts with a person under 14 constitute rape of a vulnerable person, irrespective of the young person’s stated consent or previous sexual experience. From age 14, consensual sexual activity is not prohibited solely based on age, although adolescents under 18 remain protected by laws against sexual violence and sexual exploitation [4].

The World Health Organization (WHO) defines sexual violence as any sexual act, attempt to obtain a sexual act, or other act directed against a person’s sexuality using coercion, by any person regardless of their relationship to the victim, in any setting [5]. The forced sexual intercourse reported in this study would therefore meet WHO’s definition of sexual violence and would be illegal at any age. When forced sexual intercourse occurs in a boy or girl below the age of 14, it would also fall within WHO’s definition of child sexual abuse. WHO defines child sexual abuse as the involvement of a child in sexual activity that he or she does not fully comprehend, is unable to give informed consent to, or for which the child is not developmentally prepared, or which violates the laws or social taboos of society [6]. This means that a boy or girl under 14 might report that the sexual intercourse occurred “because I wanted to,” while the experience would nevertheless meet the Brazilian legal definition of rape of a vulnerable person and could fall within WHO’s conceptualization of child sexual abuse. From age 14, consensual sexual activity is not prohibited solely because of age; however, sexual activity involving violence, coercion or sexual exploitation remains unlawful and may constitute sexual violence or sexual abuse.

There is an additional consideration in interpreting the study’s finding on forced first sexual intercourse. Participants were asked to choose whether their first intercourse occurred “because I wanted to” or “because I was forced to.” While this provides an important measure of explicitly forced intercourse, it may not capture the wider spectrum of unwanted or coerced sexual activity, including pressure, manipulation, fear, and unequal power. Research that has explored the circumstances surrounding adolescent sexual activity in greater depth has demonstrated the complex ways in which relationships, gender and social norms, economic circumstances and power inequalities can shape young people’s sexual choices and experiences [7]. A simple “wanted versus forced” dichotomy may therefore miss important dimensions of sexual agency and coercion. The 1.6% prevalence of forced first intercourse reported in the Pelotas study should be interpreted in this context.

To sum up, over six out of ten participants reported having initiated sexual activity by age 18, and among those who were sexually active, approximately one in eight did so before age 14. Early sexual initiation was associated with male sex, some sexual-orientation categories, frequent drug use, forced first intercourse and a greater number of lifetime sexual partners. It is important to note that associations with race and several social and economic factors were weak or absent after adjustment. Forced first intercourse was much less commonly reported but was strongly associated with female sex and with sexual initiation before age 14. These findings underline the importance of distinguishing between three overlapping but different issues: early sexual initiation, sexual activity below the legal age of consent, and sexual coercion or abuse.

## Implications of the study’s findings for research and action

My first recommendation relates to the finding that almost two thirds of participants had initiated sexual activity by age 18, with some doing so considerably earlier. Young people need to be prepared for the decisions and situations they may encounter as they develop sexually. They should receive comprehensive sexuality education that is scientifically accurate, age- and developmentally appropriate, and addresses relationships, consent, gender and power, prevention of sexual violence, contraception and other aspects of sexual and reproductive health. They should also have access to confidential, adolescent-responsive sexual, and reproductive health services, including contraception.

The UN International Technical Guidance on Sexuality Education provides an evidence-informed framework for delivering such education progressively from childhood through adolescence, with the aim of enabling young people to make informed, healthy and respectful choices about relationships, sexuality and reproduction [8]. WHO’s updated guideline on preventing early pregnancy and poor reproductive outcomes in adolescents in low-and-middle income countries, provides evidence-based guidance on increasing access to, uptake and continued use of contraception by adolescents [9]. The paper by da Silveira et al. provides no information on the current situation in terms of provision of/access to sexuality education and sexual and reproductive health services for young people. Informed by up-to-date information on the current situation, government authorities must take steps to ensure the provision of these services

My second recommendation relates to the findings on sexual activity below the age of 14, the legal age of consent for sexual activity in Brazil. Laws and policies intended to protect children and adolescents from sexual abuse and exploitation should not be applied in ways that unnecessarily criminalize consensual, non-exploitative sexual activity between adolescents. Age-of-consent laws should protect young people from abuse and exploitation without unnecessarily punishing them for consensual, non-exploitative sexual activity with peers of similar age and developmental status. This distinction is particularly important because criminalization may discourage adolescents from seeking information, contraception, and other sexual and reproductive health services [10,11]. WHO guidance similarly cautions against reporting consensual sexual activity between adolescents unless the adolescent’s safety is at risk [6].

A recent judgment of the High Court of Kenya provides an important perspective on this issue. In holding unconstitutional, the blanket criminalization of consensual, non-coercive and non-exploitative sexual activity between adolescents of similar ages, the Court sought to balance protection from sexual violence and exploitation with adolescents’ dignity, autonomy and evolving capacities. Importantly, the Court treated adolescents not simply as children requiring protection, but as developing individuals who are themselves rights-holders entitled to constitutional protections, including dignity, equality, privacy and access to health [10]. As Bhatia notes in his commentary on the judgment, a constitutional approach should respect rather than “infantilize” adolescents, while continuing to protect them vigorously where coercion, exploitation, abuse or power imbalances are present [10]. Addressing the Indian context, Parmar and Rathod similarly call for a more balanced age-of-consent framework that protects adolescents from abuse while respecting their evolving autonomy, through close-in-age exemptions, confidential access to sexual and reproductive health services, sexuality education that addresses consent and healthy relationships, discretionary authority in mandatory reporting, aligning laws and policies affecting adolescent sexuality with social welfare schemes, and revising judicial and prosecutorial guidelines to make them child friendly [12]. The paper by da Silveira et al. provides no information on the current situation in this area. Informed by up-to-date information on the current situation, government authorities must take steps to define, communicate, and monitor what happens on the ground.

My third recommendation relates to the findings on forced sexual activity. Every effort should be made to identify sexual activity involving coercion, abuse or exploitation, particularly among younger adolescents. This requires health-care providers and others who work with adolescents to be able to recognize possible sexual abuse, inquire about it sensitively and respond appropriately when it is disclosed. WHO recommends a child- and adolescent-centered response that prioritizes safety, privacy and confidentiality; listens to and respects the wishes of the young person; minimizes further trauma; and provides appropriate medical, psychological and social support. Where required by law, appropriate reporting and referral to child-protection and other relevant authorities should follow, while taking account of the safety and best interests of the young person [6,13].

As discussed above, the measures used by da Silveira et al. may have captured only part of the spectrum of unwanted and coerced sexual activity. Under-reporting because of shame and stigma is also likely [5,6,7]. Given this, the response to the findings on forced sexual activity must go beyond care and support for those who report that they have experienced it in this study. Legal protection and accountability are essential, but they must be accompanied by evidence-based educational, community and psychosocial interventions that address the conditions in which sexual violence occurs [14].

## Conclusion

The findings of this study point to the need for a balanced response to adolescent sexual activity. Young people need the knowledge and services that will enable them to make informed and safe choices about their sexuality; they should not be unnecessarily criminalized for consensual sexual activity; and they must be vigorously protected from coercion, abuse and exploitation. The challenge for families, schools, health services and the law is to achieve these three objectives together: to equip adolescents, avoid unnecessarily criminalizing them, and protect them from sexual violence.

## Data availability statement

N/A.

## Conflicts of interest

The author declares no conflicts of interest.
